# Case Report: Tuberculosis lymphadenitis with systemic lupus erythematosus in a young woman: a case report

**DOI:** 10.12688/f1000research.135076.1

**Published:** 2023-06-29

**Authors:** Yunita Arliny, Dewi Behtri Yanifitri, Wilia Aprilisa Utami, Samantha Geraldine

**Affiliations:** 1 Pulmonology and Respiratory Medicine, Rumah Sakit Umum Daerah Dr Zainoel Abidin, Banda Aceh, Aceh, Indonesia; 2 Pulmonology and Respiratory Medicine, Universitas Syiah Kuala, Banda Aceh, Aceh, Indonesia; 3 Faculty of Medicine, Universitas Sebelas Maret, Surakarta, Central Java, Indonesia

**Keywords:** Tuberculosis, TB lymphadenitis, Tuberculous lymphadenitis, Systemic Lupus Erythematosus, SLE

## Abstract

**Background**: Tuberculosis is a chronic infectious disease and can be categorised into pulmonary TB and extra-pulmonary TB based on its spread. TB lymphadenitis is one of the extra-pulmonary TB diseases. Patients with a weakened immune system in systemic lupus erythematosus (SLE) have an increased incidence of TB.

**Case**: Here we present a case report of a 21-year-old female patient with SLE diagnosed with tuberculous lymphadenitis at dr. Zainoel Abidin Hospital, Banda Aceh, Indonesia. The patient complained of a lump in the right neck 4 months ago with a diameter of 4 cm, accompanied by fever, decreased appetite, and weight loss. Other than that, the patient also experiences joint pain, hair loss and sun sensitivity since 12 months ago. Chest radiography showed no abnormalities, and fine-needle aspiration biopsy results confirmed tuberculous lymphadenitis. Antinuclear antibody test was borderline. The patient had been taking steroids and hydroxychloroquine for the past 10 months. Currently, the patient is taking the advanced phase of antituberculosis drugs FDC. After undergoing the intensive phase of antituberculosis drugs, the submandibular lump got smaller to a diameter of 2 cm.

**Conclusion**: TB lymphadenitis is a rare case but can occur in conditions of decreased immunity like SLE. It involves some of the immune disorders caused by the long-term use of immunosuppressive therapy.

## Introduction

Tuberculosis is a chronic infectious disease caused by the bacterium *Mycobacterium tuberculosis.* ^1^ Tuberculosis (TB) is still a major global health problem and remains one of the most common infectious diseases globally. It is estimated 8.6 million people suffered from TB in 2012 and it resulted in 1.3 million deaths globally. ^2^ Indonesia placed second in the country with the most TB cases in the world. ^3^ In its distribution, tuberculosis can be categorized into two parts: pulmonary TB and extra-pulmonary TB. Extrapulmonary TB accounts for about 15–20% of all TB cases. One example of extra-pulmonary TB disease is TB Lymphadenitis or are known as lymph node TB. Tuberculous lymphadenitis is seen in almost 35% of cases of extrapulmonary TB. ^4^ It can affect all ages, especially those aged 10–30 years, and more often in women. Enlarged lymph nodes are most commonly seen in the neck area and sometimes in the armpit area. ^5^ The lessened immune system in systemic lupus erythematosus (SLE) can increase the incidence of TB. It is suspected that inherent immunodeficiency states in SLE and the use of immunosuppressant agents as the treatment of SLE increased susceptibility to TB. The risk of TB in patients with autoimmune diseases has increased compared to patients without autoimmune diseases. ^2^ In the following, we report a case of tuberculous lymphadenitis with systemic lupus erythematosus in a young woman. ^5^

## Case description

A 21-year-old Aceh female student, came to the TB-DOTS clinic at dr. Zainoel Abidin Hospital, Banda Aceh, Indonesia with chief complaints of a lump on the right neck since 4 months ago with a diameter of 4 cm, characterized as a supple, mobile and painless lump. The patient has not complained of cough or has a history of long-standing cough. There is also no shortness of breath, chest pain, or night sweats. However, the patient has a history of fever and experienced weight loss of about 15 kg within 3 months and decreased appetite. The patient also complained of joint pain, especially in cold weather, large amounts of hair loss, feeling tired quickly to tremors when she was tired, and her skin turning red to blackish colour quickly when exposed to the sun. The patient was sent by a rheumatologist and was diagnosed with SLE 12 months ago. The patient has a history of taking hydroxychloroquine and methylprednisolone from 10 months ago. Family history showed the patient’s mother’s sister is also a patient with SLE.

**Figure 1.**
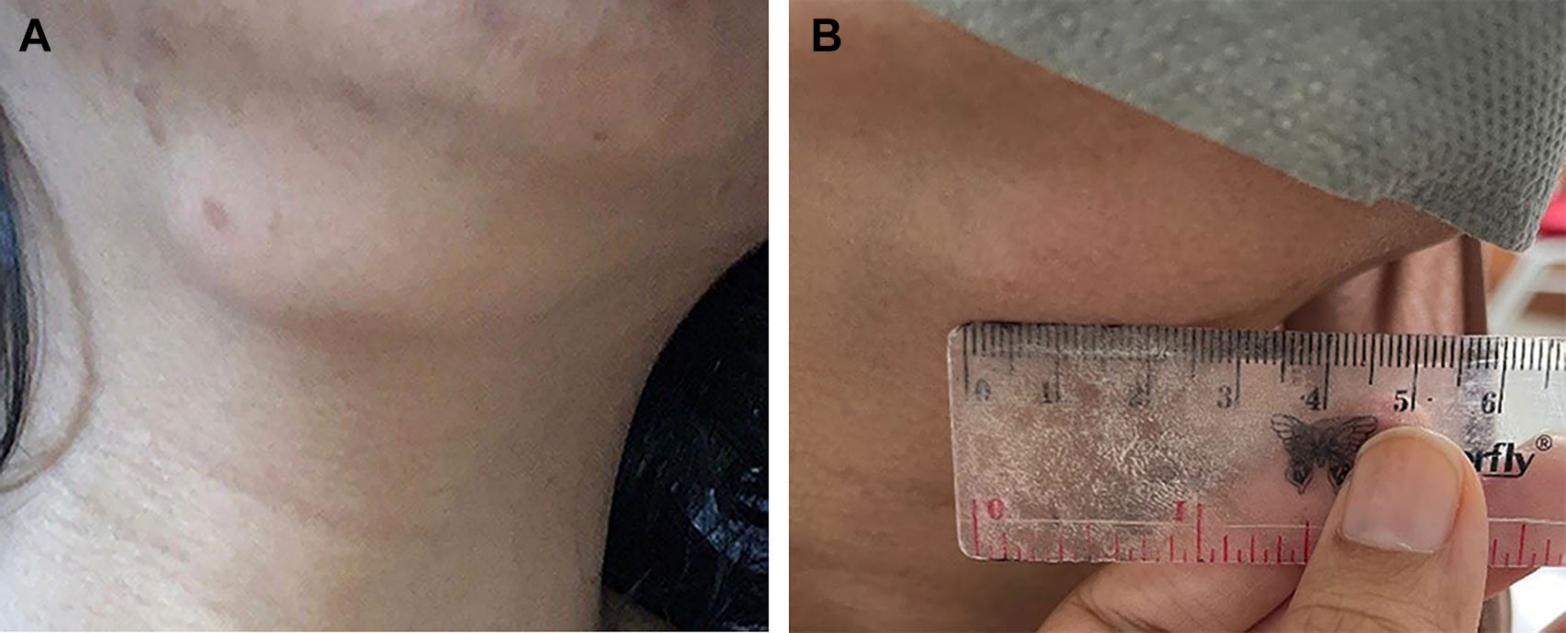
A) Photo before antituberculosis treatment (size 4×2 cm). B) Photo after the intensive phase of antituberculosis size 2×1 cm.

On examination, vital signs showed *compos mentis* consciousness and stable hemodynamically. Physical examination ( Figure 1A) palpable enlarged lymph node in the right submandibular, 4×2 cm in diameter, less defined, mobile, firm, supple, and painless since 4 months ago. After the patient took 4 tablets of FDC as antituberculosis drugs in the intensive phase, the size of the lymph node in the right submandibular decreased with a diameter of 2×1 cm ( Figure 1B). Pulmonary physical examination was normal. The results of laboratory examinations showed hemoglobin 9.9 g/dL, leukocytes 7.59/mm ^3^, platelets 381 10 ^3^/mm ^3^, urea 11 mg/dL, creatinine 0.55 mg/dL, negative rheumatoid factor (RF) and normal urinalysis results.

**Figure 2.**
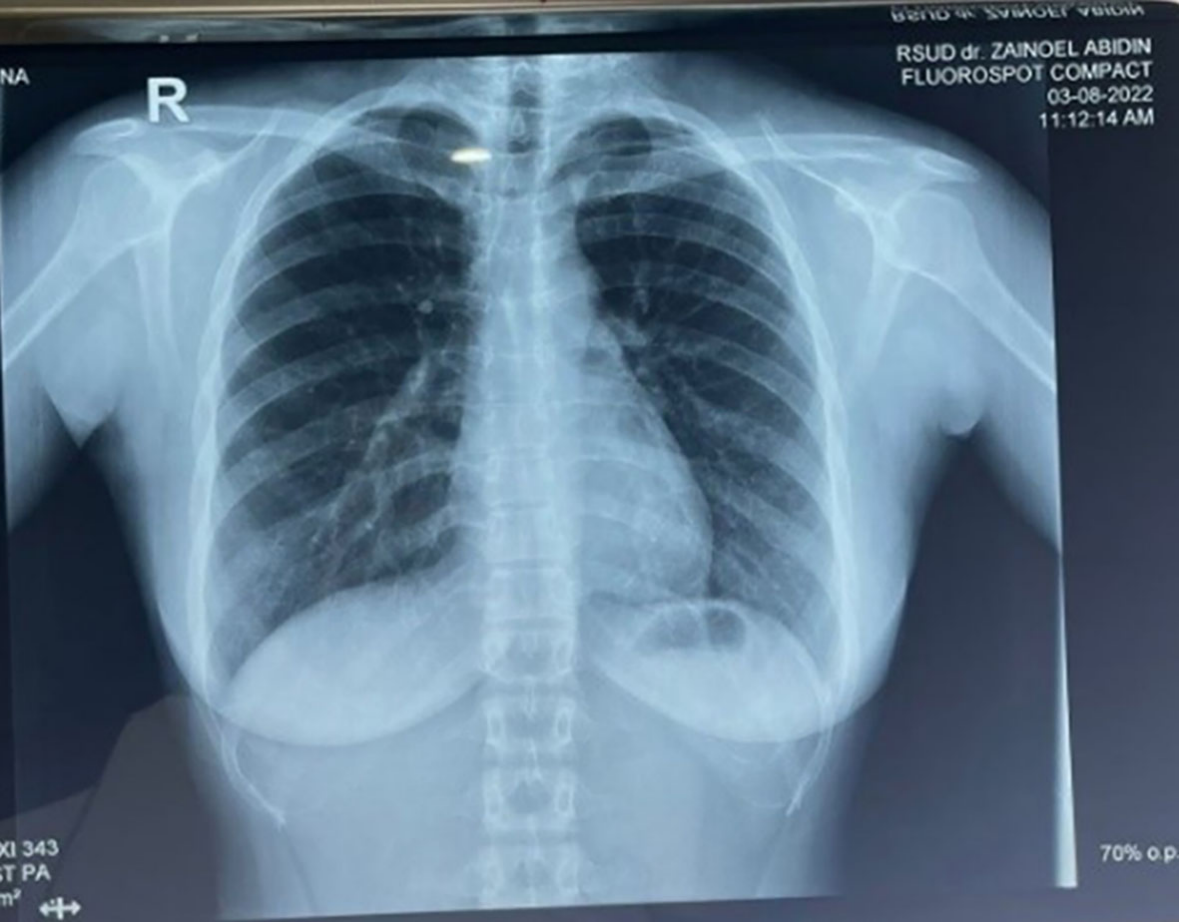
Results of thoracic photos.

Radiological examination ( Figure 2) showed normal lung results. The FNAB examination of the submandibular lump showed epithelioid cells between the reticular fibres, and the smear background consisted of minimal red blood cells with the conclusion being suggested to chronic lymphadenitis that is commonly found in tuberculosis infection.

The working diagnosis in this patient was TB lymphadenitis and planned to give the patient antituberculosis drugs for 9 months, divided into 2 months of the intensive phase and 7 months of the continuation phase. The intensive phase started in July 2022, the patient got antituberculosis drugs, 3 tablets of 4 FDC/day. In October 2022, the patient started the continuation phase and got antituberculosis drugs, 3 tablets of 2 FDC/day. We added vitamin B6 100 mg once a day for the treatment. The patient had previously been diagnosed with SLE from the results of the ANA test that have been checked in November 2021, which indicated a borderline result. When she was first treated at the rheumatology polyclinic, the patient complained of joint pain, frequent weakness, and hair loss, and her skin turned red quickly when exposed to sunlight, which she had complained about 12 months ago. The rheumatologist has given therapy hydroxychloroquine 1×200 mg and methylprednisolone 2×4 mg. After the patient was diagnosed with TB lymphadenitis 3 months ago, the rheumatologist stopped methylprednisolone therapy and the patient is now only taking hydroxychloroquine 1×200 mg from the rheumatology doctor. By this time the patient had completed the entire phase of antituberculosis drugs. During the evaluation after 9 months of antituberculosis drugs, the size of the lymph node decreased and the patient had no complaints.

## Discussion

Based on the anamnesis, physical examination and supporting examination, the patient was diagnosed with TB lymphadenitis. The general symptoms between tuberculosis lymphadenitis and pulmonary tuberculosis are the same. The symptoms that can always be found include sub febrile, decreased appetite, weight loss, weakness, and night sweats. The physical appearance of superficial TB lymphadenitis is classified into 5 stages by Jones and Campbell, namely: (a) Stage I: Enlarged lymph nodes with a spongy consistency, mobile/easy to move, separated from other nodules, this indicates a nonspecific hyperplastic reaction; (b) Stage 2: larger than stage 1 with a spongy consistency, adherent to the surrounding tissue/confluent; (c) Stage 3: central tenderness due to abscess formation; (d) Stage 4: collar stud abscess formation/redness over the abscessed skin; (e) Stage 5: formation of sinuses that drain purulent secretions. ^6^ As noted in this case the patient was stage 1. One of the difficulties in establishing a clinical diagnosis of TB lymphadenitis is the absence of gene expert results from tissue samples, so we can only diagnose based solely on the patient’s clinical condition.

**Figure 3.**
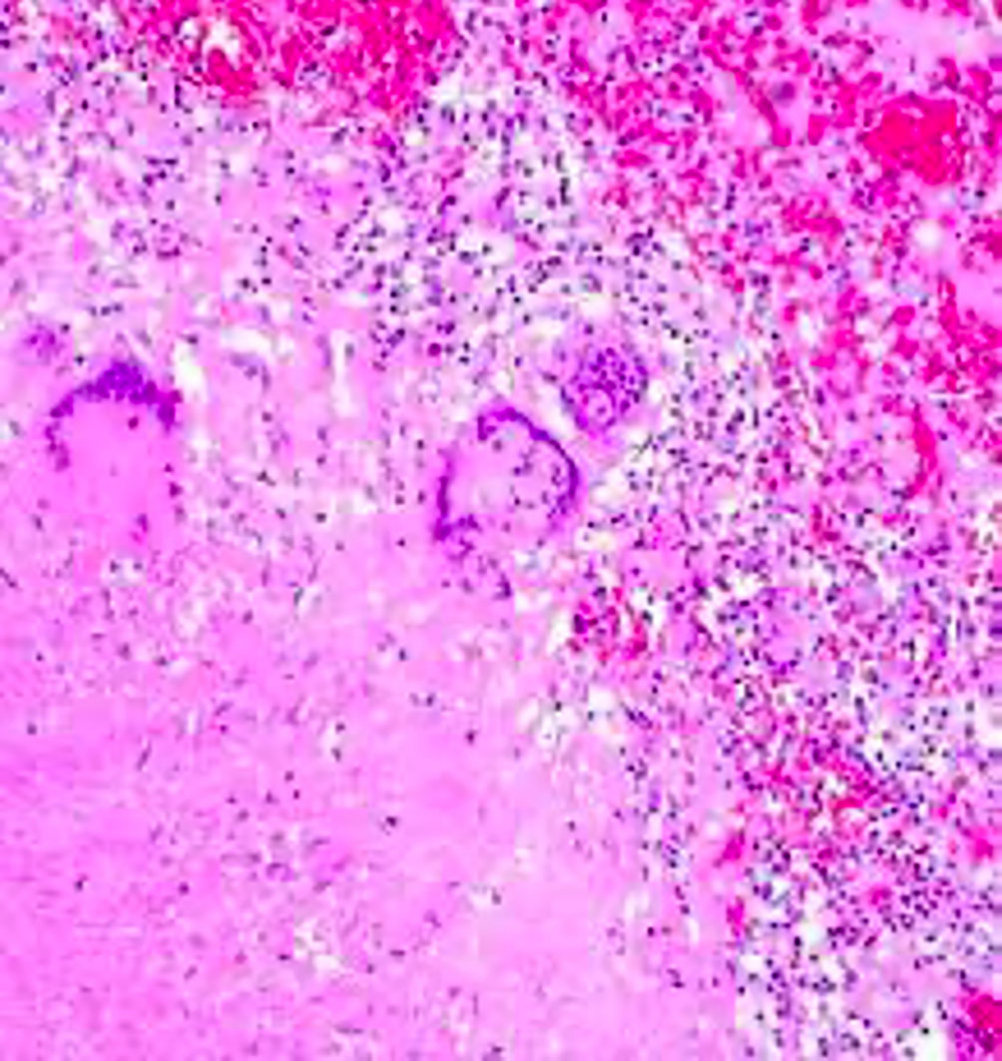
Granulomatous inflammation.

Fine needle aspiration biopsy (FNAB) is a simple, safe, and inexpensive procedure for diagnosing TB lymphadenitis. In addition, it has high sensitivity (78.95%) and specificity (90.32%) values. ^7^ Epithelioid morphology ( Figure 3) with necrosis was found in all TB cases in the study by Suryadi, 2020, which occurred due to inflammation and tissue damage caused by stimulation macrophages to eradicate mycobacteria so that the Th1 response leads to the formation of granulomas and necrosis. Activated macrophages can form cytoplasm that resembles many epithelial cells called epithelioid cells. ^8^

Systemic lupus erythematosus (SLE) is an autoimmune disease whose cause is unknown with very diverse clinical features, so it is often referred to as a disease with a thousand faces. ^9^ The most common symptoms of SLE are in the musculoskeletal system, in the form of arthritis or arthralgia (93%) often precedes other symptoms. Disorders of the skin, hair or mucous membranes are found in 85% of cases of SLE, and kidney disorders are found in 68% of cases of SLE. ^10^ This disease is more common in women than men with a ratio of 12:1 and can affect all ages. Based on research by Kasjmir et al., genetic, gender, and environmental factors are thought to be factors that influence SLE. Early diagnosis of SLE is not easy because the course of the disease is very varied and often manifests as other diseases. ^11^

Autoimmune diseases including SLE, especially where immunosuppressive drugs are required based on disease activity, can increase the risk of TB. In SLE patients there is an abnormality of the immune system and the patient is receiving an immunosuppressant agent as therapy. This condition causes the patient to have a high risk of infection, one of which is TB infection. This has also become the major risk factor that accounts for the high prevalence of TB infection in SLE. ^12^ A cohort study on the incidence of TB in SLE patients in Indonesia showed a higher incidence in patients diagnosed with SLE under the age of 25, just like the patient in this report. The study also showed that the median time of being diagnosed with TB was 2 years after being diagnosed with SLE. ^13^ Corticosteroids and immunosuppressive agents are the mainstays of treatment in SLE. A retrospective case–control study in Colombia showed that 12 months of cumulative steroid doses of 1830 mg nearly tripled the risk of TB. ^14^ A 13-year cohort study of SLE patients in Taiwan has shown that corticosteroid treatment is associated with a more than 10-fold increased risk of TB. ^15^ It is in line with our study where the patient was diagnosed with SLE and took an immunosuppressant agent before the development of the lump that was suspected as TB lymphadenitis. This case is also in accordance with research by Hamijoyo et al. and Damara et al. Those with the most extrapulmonary TB manifestations were tuberculous lymphadenitis followed by miliary TB and TB meningitis. ^13^^,^^16^

In general, the management of extra-pulmonary TB is divided into medical therapy and surgical therapy. Medical therapy uses first-line anti-TB drugs as the main therapy. Antituberculosis drugs are given using the same regimen as in pulmonary TB therapy, but with a longer treatment period of 9 months. ^17^ SLE therapy with hydroxychloroquine and chloroquine is recommended as initial therapy for mild or moderate SLE, for example in skin rashes and arthritis. It is currently recommended that all patients with active SLE be given antimalarials, hydroxychloroquine has a lower chance of kidney damage compared to patients who do not receive it. ^18^ For a suitable surgical approach for cervical tuberculous lymphadenitis, we need to consider the characteristics and location of the lesion, along with CT imaging and manifestations during presurgical evaluation. In closing, this case report is a case from a tertiary hospital that does not yet have complete registered follow-up data and incomplete bacteriological examination, including TB gene experts from sputum and biopsy tissue, however, it still provides comprehensive information that leads to increased awareness of TB in SLE patients. It is necessary to improve screening and preventive therapy for latent TB so that the risk of developing active TB can be minimized.

## Conclusion

In SLE patients with high-dose steroid treatment and clinical infection that does not improve, it is necessary to consider the presence of TB infection, especially extrapulmonary TB, like lymphadenitis. The risk patients with immunocompromised conditions like SLE and getting immunosuppressant agents as therapy can increase the risk of developing TB infection 7 times more compared to the general population.

## Consent to participate

Informed written consent was obtained from the patient for the publication of this report and any accompanying images.

## Data Availability

All data underlying the results are available as part of the article and no additional source data are required.
