# Supplementary material for: Case Report: Tuberculosis lymphadenitis with systemic lupus erythematosus in a young woman: a case report
Source: F1000Res. 2023 Oct 16;12:763. Originally published 2023 Jun 29. [Version 2] doi: 10.12688/f1000research.135076.2 (PMC10643883; doi:10.12688/f1000research.135076.2)
Supplement: Peer review history

# Reviewer report: Reviewer response for version 2

**DOI:** 10.5256/f1000research.157467.r215699

**Published:** 2023-11-13

**Authors:** PB Sryma

**Affiliations:** 1 Department of Pulmonary, Critical Care and Sleep Medicine, All India Institute of Medical Sciences (AIIMS), New Delhi, Delhi, India

The edited article can be accepted for indexing as is. Changes have been made according to the suggestions.

Are enough details provided of any physical examination and diagnostic tests, treatment given and outcomes?

Yes

Is the case presented with sufficient detail to be useful for other practitioners?

Partly

Is sufficient discussion included of the importance of the findings and their relevance to future understanding of disease processes, diagnosis or treatment?

Partly

Is the background of the case’s history and progression described in sufficient detail?

Yes

Reviewer Expertise:

NA

I confirm that I have read this submission and believe that I have an appropriate level of expertise to confirm that it is of an acceptable scientific standard.

# Reviewer report: Reviewer response for version 2

**DOI:** 10.5256/f1000research.157467.r215698

**Published:** 2023-10-31

**Authors:** Gyanshankar Mishra

**Affiliations:** 1 Indira Gandhi Government Medical College, Nagpur, Maharashtra, India

The case report, reports Tuberculosis lymphadenitis with systemic lupus erythematosus in a young woman. A significant contribution to the literature and highlights the importance of suspecting TB in immunocompromised conditions.

Are enough details provided of any physical examination and diagnostic tests, treatment given and outcomes?

Yes

Is the case presented with sufficient detail to be useful for other practitioners?

Yes

Is sufficient discussion included of the importance of the findings and their relevance to future understanding of disease processes, diagnosis or treatment?

Yes

Is the background of the case’s history and progression described in sufficient detail?

Yes

Reviewer Expertise:

Respiratory Diseases, Tubercuolosis

I confirm that I have read this submission and believe that I have an appropriate level of expertise to confirm that it is of an acceptable scientific standard.

# Reviewer report: Reviewer response for version 1

**DOI:** 10.5256/f1000research.148177.r205699

**Published:** 2023-10-31

**Authors:** Henna Iqbal

**Affiliations:** 1 Mercer University School of Medicine, Macon, GA, USA

This case only diagnosed TB on the basis of histological findings of a lymph node, while no other confirmatory tests were performed. It was fortunate to have a response to treatment, but ethically, anti-TB drugs should not be prescribed on suspicion only. There should be a confirmatory culture or molecular assay to certainly say this was tuberculosis. Technically, anti-inflammatory effect on antibiotics can also work in favor. SLE was also similarly diagnosed just on basis of ANA, no SLE specific antigen was found. This could have been discoid lupus if skin hypersensitivity was the only striking feature.

Are enough details provided of any physical examination and diagnostic tests, treatment given and outcomes?

No

Is the case presented with sufficient detail to be useful for other practitioners?

Partly

Is sufficient discussion included of the importance of the findings and their relevance to future understanding of disease processes, diagnosis or treatment?

No

Is the background of the case’s history and progression described in sufficient detail?

Partly

Reviewer Expertise:

microbiology

I confirm that I have read this submission and believe that I have an appropriate level of expertise to state that I do not consider it to be of an acceptable scientific standard, for reasons outlined above.

# Response

**Published:** 2023-10-31

**Authors:** Yunita Arliny

**Affiliations:** Pulmonology and Respiratory, Universitas Syiah Kuala, Banda Aceh, Aceh, Indonesia

Dear Reviewer,

 Thank you for your review. 

 Here we present you the clarification based on guidelines from the Indonesian Ministry of Health about the diagnostic of TB Lymphadenopathy.

 “Tuberculosis lymphadenopathy can affect all ages, especially

 at the age of 10-30 years, more often in women. Constitutional symptoms

 occur in 33-85% of patients. Diagnosis is made through needle aspiration smooth or gland biopsy. Preparations from fine needle aspiration or Biopsy can reveal caseous granulomatous inflammation with cells Datia Langhans. The diagnosis can ALSO be confirmed through the TB rapid molecular test.”

 So, needle aspiration can be a diagnostic method as in our article with or without molecular tests to confirm the diagnostic.

 If you have any other suggestions, please don’t hesitate.

 Thank you.

 Regards,

 Yunita Arliny

# Reviewer report: Reviewer response for version 1

**DOI:** 10.5256/f1000research.148177.r205688

**Published:** 2023-10-12

**Authors:** Gyanshankar Mishra

**Affiliations:** 1 Indira Gandhi Government Medical College, Nagpur, Maharashtra, India

This case report underscores the critical clinical consideration of tuberculosis suspicion in autoimmune patients undergoing immunosuppressive therapy. The prudent application of timely tuberculosis preventive therapy before the commencement of immunosuppressive treatment is a rational approach for such patient populations.

 A grammatical review and corrections are warranted for the manuscript. The following are suggested:

**Abstract:**

- The case report should employ past tense: e.g. "In addition, the patient had been experiencing joint pain, hair loss, and sun sensitivity for the past 12 months." Change: "Currently, the patient is taking the advanced phase of antituberculosis drugs FDC" to "Currently, the patient is undergoing the continuation phase of antituberculosis drugs FDC."
- Conclusion: "TB lymphadenitis is a rare case but can occur in conditions of decreased immunity like SLE. It involves some of the immune disorders caused by the long-term use of immunosuppressive therapy." Change to: "TB lymphadenitis can occur in conditions of decreased immunity, particularly in cases of SLE, especially when patients are on long-term immunosuppressive or corticosteroid therapy."
- TB lymphadenitis is not a rare disease; as mentioned in the introduction section of the case report, it comprises 35% of extrapulmonary TB cases.

**Case report:**

- Introduction: "In 2012, an estimated 8.6 million people suffered from TB, resulting in 1.3 million deaths globally. Indonesia ranked second among countries with the highest TB cases in the world." The World Health Organization's Global Tuberculosis Report for 2016 has been referenced. Please update the statistics to refer to the World Health Organization's Global Tuberculosis Report for 2023.
- The case report part can be rewritten as follows:
  Case report: "The patient did not complain of cough symptoms and had no history of chronic cough. Furthermore, there were no manifestations of dyspnea, chest pain, or nocturnal sweats. However, the patient had a documented history of fever and experienced a weight loss of approximately 15 kg over the course of three months, accompanied by a notable decrease in appetite. The patient also complained of joint pain, particularly exacerbated in cold environmental conditions, substantial hair loss, rapid onset of fatigue leading to tremors when fatigued, and a tendency for her skin to undergo rapid erythematous and sometimes hyperpigmented changes upon sun exposure. Notably, the patient was referred by a rheumatologist and was diagnosed with Systemic Lupus Erythematosus (SLE) 12 months ago. The patient had been on a therapeutic regimen incorporating hydroxychloroquine and methylprednisolone for the past 10 months. Additionally, the patient's family history revealed that her maternal aunt also had a confirmed diagnosis of SLE."
- Change: "Figure 2. Results of thoracic photos" to "Chest radiograph PA view."
- Change: "Radiological examination (Figure 2) showed normal lung results" to "The chest radiograph was normal."
- Change: "The FNAB examination of the submandibular lump showed epithelioid cells between the reticular fibres, and the smear background consisted of minimal red blood cells with the conclusion being suggested to chronic lymphadenitis that is commonly found in tuberculosis infection" to "The Fine Needle Aspiration Biopsy (FNAB)...".
- Discussion and conclusion align with the case report.

Overall the article is a useful addition to medical literature

Are enough details provided of any physical examination and diagnostic tests, treatment given and outcomes?

Yes

Is the case presented with sufficient detail to be useful for other practitioners?

Yes

Is sufficient discussion included of the importance of the findings and their relevance to future understanding of disease processes, diagnosis or treatment?

Yes

Is the background of the case’s history and progression described in sufficient detail?

Yes

Reviewer Expertise:

Respiratory Diseases, Tubercuolosis

I confirm that I have read this submission and believe that I have an appropriate level of expertise to confirm that it is of an acceptable scientific standard, however I have significant reservations, as outlined above.

# Reviewer report: Reviewer response for version 1

**DOI:** 10.5256/f1000research.148177.r193131

**Published:** 2023-08-10

**Authors:** PB Sryma

**Affiliations:** 1 Department of Pulmonary, Critical Care and Sleep Medicine, All India Institute of Medical Sciences (AIIMS), New Delhi, Delhi, India

The article is well written, constructed well and organised. Discussion is well written with detailed review of literature.

**Minor comments:**

- It will be better if the authors can provide any data regarding microbiological confirmation of tuberculosis and describe the histopathology with presence of necrotising granulomas if they were present.

Are enough details provided of any physical examination and diagnostic tests, treatment given and outcomes?

Yes

Is the case presented with sufficient detail to be useful for other practitioners?

Partly

Is sufficient discussion included of the importance of the findings and their relevance to future understanding of disease processes, diagnosis or treatment?

Partly

Is the background of the case’s history and progression described in sufficient detail?

Yes

Reviewer Expertise:

ARDS, Tuberculosis, sarcoidosis, mediastinal lymphadenopathy

I confirm that I have read this submission and believe that I have an appropriate level of expertise to confirm that it is of an acceptable scientific standard, however I have significant reservations, as outlined above.

# Response

**Published:** 2023-08-10

**Authors:** Yunita Arliny

**Affiliations:** Pulmonology and Respiratory, Universitas Syiah Kuala, Banda Aceh, Aceh, Indonesia

Dear Reviewer,

 Thank you for the input, but we have some questions and confirmation regarding this.

 1. We already wrote the FNAB result in the text. Can you elaborate more on what you mean by the histopathology result?

 2. The patient was confirmed to have TB lymphadenitis based on the FNAB result. We didn't do the microbiology test from sputum because the patient didn't show the TB sign and symptoms and the x-ray result was clear.

 We're looking forward to your reply.

 Thank you.

 Regards, 

 Yunita Arliny
